# Designing assisted living technologies ‘in the wild’: preliminary experiences with cultural probe methodology

**DOI:** 10.1186/1471-2288-12-188

**Published:** 2012-12-20

**Authors:** Joseph Wherton, Paul Sugarhood, Rob Procter, Mark Rouncefield, Guy Dewsbury, Sue Hinder, Trisha Greenhalgh

**Affiliations:** 1 Centre for Primary Care and Public Health, Barts and the London School of Medicine and Dentistry, London, UK; 2 Newham University Hospital, Barts Health NHS Trust, London, UK; 3 Manchester e-Research Centre, Arthur Lewis Building, University of Manchester, Manchester, UK; 4 Lancaster University, Lancaster, UK; 5 gdewsbury, gdewsbury.com

**Keywords:** Qualitative research, Cultural probes, Assistive technology, Ethnography

## Abstract

**Background:**

There is growing interest in assisted living technologies to support independence at home. Such technologies should ideally be designed ‘in the wild’ i.e. taking account of how real people live in real homes and communities. The ATHENE (Assistive Technologies for Healthy Living in Elders: Needs Assessment by Ethnography) project seeks to illuminate the living needs of older people and facilitate the co-production with older people of technologies and services. This paper describes the development of a cultural probe tool produced as part of the ATHENE project and how it was used to support home visit interviews with elders with a range of ethnic and social backgrounds, family circumstances, health conditions and assisted living needs.

**Method:**

Thirty one people aged 60 to 98 were visited in their homes on three occasions. Following an initial interview, participants were given a set of cultural probe materials, including a digital camera and the ‘Home and Life Scrapbook’ to complete in their own time for one week. Activities within the Home and Life Scrapbook included maps (indicating their relationships to people, places and objects), lists (e.g. likes, dislikes, things they were concerned about, things they were comfortable with), wishes (things they wanted to change or improve), body outline (indicating symptoms or impairments), home plan (room layouts of their homes to indicate spaces and objects used) and a diary. After one week, the researcher and participant reviewed any digital photos taken and the content of the Home and Life Scrapbook as part of the home visit interview.

**Findings:**

The cultural probe facilitated collection of visual, narrative and material data by older people, and appeared to generate high levels of engagement from some participants. However, others used the probe minimally or not at all for various reasons including limited literacy, physical problems (e.g. holding a pen), lack of time or energy, limited emotional or psychological resources, life events, and acute illness. Discussions between researchers and participants about the materials collected (and sometimes about what had prevented them completing the tasks) helped elicit further information relevant to assisted living technology design. The probe materials were particularly helpful when having conversations with non-English speaking participants through an interpreter.

**Conclusions:**

Cultural probe methods can help build a rich picture of the lives and experiences of older people to facilitate the co-production of assisted living technologies. But their application may be constrained by the participant’s physical, mental and emotional capacity. They are most effective when used as a tool to facilitate communication and development of a deeper understanding of older people’s needs.

## Background

### The ATHENE project

Aging of the population in many countries is fuelling interest in assisted living technologies (ALTs) to support independence at home. This includes *telecare* (continuous and remote monitoring of real time emergencies and lifestyle changes through sensor devices and personal trigger alarms) and *telehealth* (transmission of medical data and information over telecommunication technologies to deliver health-related services to the home), which are designed to deliver better and more cost effective social and health care into the home. Many diseases (e.g. diabetes, dementia, arthritis) increase in prevalence with age, as does comorbidity. Some epidemiologists predict that demand will soon outstrip supply for essential medical services [1,2]. There is much policy interest in ‘information-age’ medicine (supported self-management by ‘empowered’ patients with professionals in a guiding role) [3], though research into the use of assisted living technologies by older people with multiple comorbidities to relieve pressure on conventional health services has shown mixed findings [4,5]. The home environment is private and potentially sensitive. An understanding of the technological opportunities and requirements presented by domestic settings is critical for the development of new solutions (technologies and ways to support their use) that meet the needs and wishes of the older person and which are also acceptable and affordable by their family and health and social care providers.

The ATHENE (Assistive Technologies for Healthy Living in Elders: Needs Assessment by Ethnography) project, funded by the UK Technology Strategy Board under its Assisted Living Innovation Platform, is using ethnographic methods to illuminate the daily living needs of older people [6]. The overarching goal is to produce a richer understanding of the complex and diverse living experiences and needs of older people and how industry, the National Health Service (NHS), social services and third sector can work with older people to ‘co-produce’ useful and useable ALTs.

ALTs do not operate in isolation. Their uptake and use are mediated by features and routines in the domestic setting, as well as wider social factors relating to the family, community and public sector services. This means that the development of solutions must be grounded in the lived experiences of users. The integration of new technologies and existing domestic routines also requires a mutually adaptive and evolving process. Co-production (or co-realisation) is a methodology for delivering innovation which focuses on user-centred, ‘design-in-use’ of both technology and practices through continually feeding back users’ experiences into ongoing design and development [7,8].

The ATHENE project consists of two phases. Phase one involves ethnographic studies of 30-50 individual cases to map the complex healthcare, social care and socio-cultural needs of older people and their carers from a range of ethnic and social groups. Phase two takes forward up to 10 volunteer cases to explore how older people and their families can work directly with industry designers to produce fit-for-purpose technologies, or adapt existing technologies, that fit in with people’s lives and lifestyles. The project will inform new ways for conducting the co-production of ALTs and identify effective ways of involving older people from different ethnic and cultural groups.

### Cultural probes

Pursuing ethnography in domestic settings raises practical and ethical challenges. Cultural probes offer a relatively unobtrusive way of providing insight into how technology could fit (and why it sometimes does not fit) into a particular home environment. The cultural probe method includes open-ended and evocative activities for participants to pursue in their own time to help narrate and depict their lives to researchers and technology designers. It applies digital cameras, dictaphones, diaries and other artefacts [9]. This approach has been used fairly extensively in design research, and has begun to be applied in domestic settings for non health-related design projects where access for conventional observational study methods is problematic. To date, cultural probes have had limited use in a healthcare context.

Gaver et al. first developed the cultural probe method as part of a collaborative approach to designing innovative technologies for the home [9]. They used a range of materials for participants to record their daily lives in order to inspire creative responses among a design team. The cultural probe packages included local and world maps, postcards, disposable camera, photo album and a diary. These were given to participants to use and return after a period of time. For example, the postcards included questions related to attitudes towards their lives and technology, which participants could respond to and post back. The disposable cameras included suggestions for photographs written on the back (e.g. ‘what you will wear today’, ‘something desirable’ and ‘something boring’). The maps could be used to mark zones showing where the person had been, where they met people, where they would like to go (but perhaps could not), and so on. Responses were used in an open-ended way to inspire design ideas.

Cultural probes have subsequently been used in different ways with different affordances (properties of objects that enable or invite certain actions or uses). These have been described variously as ‘technology probes’, ‘domestic probes’, ‘mobile probes’, ‘cognitive probes’, and ‘informational probes’ [10-12]. Different research groups interpreted the data from such probes in different ways. Because Gaver et al. used probes as inspirational tools for design teams, the value of these probes was considered to lie in the uncertainty and subjective interpretation of the materials produced. The intention was not to identify a specific set of problems or technological requirements, as would occur in a formal design specification, but instead to capture in a more general way the users’ “beliefs and desires, their aesthetic preferences and cultural concerns” ( [9], page 25). Others have used cultural probe methods to help build a richer understanding of participants’ perspectives and experiences [11].

Despite differences in style and interpretation, at a more theoretical level, cultural probes have a number of common features [10,11]. First, they serve as ‘capture artefacts’ with affordances for data collection. For example, cameras, voice recorders, maps, diagrams and postcards all encourage image-rich responses from the participant. Second, they support collection of data in autobiographical (narrative) format, documenting fragments of people’s lives and providing insight into their life stories and daily routines. Narratives emerge, especially when the participant and researcher review the probe materials together, as the person gives an account of how a photograph came to be taken or elaborates on an event recorded in their diary. Third, probes can ‘make the familiar strange’, by capturing mundane and everyday actions, places, objects and people. Fourth, probes can add personal meaning and significance to data by recording such things as wishes, desires, emotions and intentions. Fifth, the process is inherently participatory since it includes the participant in the research process as an active contributor and ‘expert’ in his or her own life, rather than a passive subject of the study – hence addressing some ethical concerns about research on older people (but also raising other concerns, as discussed below). Finally, cultural probes support dialogue and conversation between researcher and participant, thereby potentially helping to overcome power imbalances between them.

Cultural probes have been criticised by some authors as lying within an ‘uncritical’ set of methodologies, comprising short-cut ethnographic tools oriented towards the superficial goal of ‘implications for design’ [13]. These tools, it is argued, distract the research gaze from the complex social and political determinants that structure and constrain human action. For example, the collection of image-rich and evocative data from an individual using cultural probes may inspire the creative imagination to produce new technologies, but this approach allegedly ignores the fact that the individual could never afford to buy the technology whose design they have inspired. The counter-argument is that cultural probes lend themselves to both ‘superficial’ and ‘critical’ applications of the ethnographic method, and that if used reflexively and systematically they could enhance rather than suppress the critical gaze by allowing researchers to engage more fully with the individual in their family, social and political context.

The aim of this paper is to report the development and initial use of a cultural probe tool in the ATHENE project. We present examples from a sample of 31 cases to demonstrate how the probe materials supported ethnographic data collection in the home with older adults with different health conditions, family and cultural circumstances and assisted living needs. Before describing these initial findings in detail, we review previous uses of cultural probes relevant to a healthcare context.

### Deployment of cultural probes with older adults

#### Domestic environments

Leonardi et al. used cultural probes with 19 older adults to see how domestic spaces related to everyday activities and emotions [14]. The probe packs included pens, paper and sticky labels which included cues, such as *“The place where I meet friends”* and *“The place where I feel safe”*. Additionally, they were given a camera to take pictures of areas and objects in the home, a photo album to collect and organise pictures and a diary to record day-to-day events. The authors found that analysis of the materials collected helped understand the functional and emotional aspects of the home. For example, functional objects and new technologies were generally found in the kitchen, an area that was associated with activity and risk. The bedroom, on the other hand, involved little activity but included various symbolic objects and mementos. The authors pointed out the need to consider how technology can be augmented within the home given the distribution of functional and emotional roles. For example, new technological devices may be more acceptable in the kitchen, which was associated with instrumental activities and danger, than in the bedroom, which was seen as a place of comfort and privacy.

Christforetti et al. documented similar distinctions within the home environment among elderly widows [15]. Their study included tools to draw and label layouts of the home, as well as a camera, diary, and a small suitcase to collect meaningful domestic items. These revealed different emotional constructs within the home. For example, they described ‘heart displacement’, in which the centre of the home shifted from a place with emotional-affective value to a place of multi-functional value. They also described the ‘refuge’ within the home to refer to boundaries drawn between the public and the private space. The bedroom was generally considered to be the most private and secure place in the home. This study highlights the close relationship between the spatial/physical and emotional/symbolic aspects of the home which could have a bearing on the adoption of new assistive devices or equipment within the home.

Other researchers have focused specifically on the use of technology and appliances within the domestic environment [16,17]. Buchmuller et al. aimed to inform the design of a cordless telephone that met the needs and demands of older users. Cultural probe materials included a disposable camera with instructions (e.g. record locations where the handset was usually found, indicate areas where users did not want to be disturbed) [17]. The accounts provided insight into where telephone base stations were positioned (e.g. hallways and dark areas of the home), how the telephone was moved around the home (e.g. left where daily housework is carried out or buried under post and newspapers) and problems (e.g. interrupted conversations as a result of empty battery). Example design requirements emerging from these insights included well-lit displays, illuminated keypads, ring tone volume control to make it easier to find and a visual and audio indication of the battery running low.

#### Social networks and supports

Riche et al. deployed cultural probes to understand the supportive role of social networks [18]. Packs included a ‘relationship map’, in which participants placed labels of social contacts onto a set of concentric rings to indicate frequency of interaction, with most frequent contacts in the innermost ring. On the map, participants were invited to include details of each person (e.g. relationship, temporal, geographical and emotional importance). Analysis of interviews, observations and probe returns revealed the role of peer support behaviour (e.g. check-up calls and exchanging house keys for emergencies), ‘aw*areness, rhythms and routines’* (e.g. if neighbour had not left the house) and trade-offs between protecting privacy and accepting peer support. Pedell et al. focused on the role of domestic technologies in addressing social isolation [19]. They presented three older participants, identified as being socially isolated, with a Polaroid camera to take pictures related to social interaction, as well as a diary with cued phrases (e.g. “Today I feel lonely because…” or “Every day I…”). The authors described six needs of older people that emerged from a review of cultural probe materials: maintaining connections with other people, managing connections, education, reciprocity (be able to offer help as well as receive help), reminiscence, and independence.

Others have explored social interactions by incorporating probes into the everyday lives of the participants as communication tools, and observed how users reacted and utilised them. Hutchinson et al. deployed a ‘technology probe’, the MessageProbe, as a communication tool among families [20]. The probe consisted of a writable LCD tablet screen, which acted as a bulletin board for remote family members to send messages. This deployment involved ‘seeding’ technologies into families’ homes to develop new ideas. Unlike conventional technology prototypes, which typically are close in form and function to the eventual finished artefact, a technology probe is deliberately simple (with only one main function), open-ended (with respect to use) and logs usage data. The authors reported how the probe revealed communication patterns, particularly with regard to coordination of activities (e.g. picking up children) and playful ways of creating remote awareness of each other.

#### Health and assisted living

Crabtree et al. used cultural probes to explore daily living needs in sensitive care settings, including a hostel for former psychiatric patients, elderly participants living at home, and a person living with stroke [21]. The probe packs included disposable cameras, scrapbooks, ‘visitor book’, diaries, dictaphones and maps of the local area. The returns were used to prompt discussions in follow-up interviews to understand opportunities and requirements for ALTs. For example, diary entries illustrated the importance of routines related to medication and diet management, highlighting the need to minimise any disruption to existing patterns and routines when implementing technological supports. Axelrod et al. explored motivational issues among stroke patients to understand how pervasive health applications could support physiotherapist-prescribed home exercises [22]. Participants were given a set of elicitation activities in a gift box. Each item was included to prompt participants to think about motivation. Example items included ‘red letter day’ cards for recording events, a wish list, a medal to trigger stories of achievement, a swimmer toy relating to struggle, card sorting of pastimes, positive and negative affect diaries and clay for modelling motivation related ideas. Discussions around the selected items were incorporated into a broader semi-structured home visit interview and tour of the home. The artefacts triggered discussions, which the authors felt would not have emerged otherwise. They also found that the ambiguity of the tasks broadened scope for conversations related to motivation.

In sum, the literature suggests there are a number of affordances of cultural probes that offer potential for capturing data relevant to ALT design, including what matters to people and why; the lived experience, physical limitations and emotional significance of the domestic space; what the key family/social relationships are and how these play out.

## Method

### Ethics and governance of the study

The study was part of the ATHENE project, funded by the UK Technology Strategy Board under its ALIP (Assisted Living Innovation Platform) call. Ethical approval was granted from Queen Mary University of London Research Ethics Committee (QMREC2011/38 1st June 2011), Harrow NHS Research Ethics Committee (11/LO/0737, 8th July 2011) and subsequent amendments.

The in-depth nature of the study raised particular challenges. The expectation that older participants with assisted living needs would be involved in the data collection process with the probe materials could place high demands on them, though other qualitative methods (e.g. open-ended interviews, home tour) could be equally demanding to someone with limited physical energy or cognitive capacity. When introducing the Home and Life Scrapbook, we emphasised that use of the probes was optional and they were free to choose what activities they wanted to do. Allowing choice was also important for guiding the interview towards issues most meaningful to participants. Similarly, we were flexible with interviews and visits, curtailing them if participants appeared fatigued or distressed.

Different issues arose across the different cases. For example, some were unable or unwilling to take photographs or complete any part of the Home and Life Scrapbook, whereas others wished to restrict the home tour to particular rooms.

All participants provided written consent before taking part. Written consent from a family carer was also provided for recruitment of any individuals whose ability to give informed consent was partially impaired due to cognitive impairment. In such cases the family carer was present during the interviews and provided advice on the participant’s wishes and/or assistance with cultural probe activities.

All personal details and information collected during the interviews were kept confidential. The identity of the participant, and of any individual mentioned by them (e.g. family, friends, care staff) were also annonymised. Data was pseudonymised at an early stage of data management and fictitious names used throughout publications and presentations.

### Participants

Thirty one participants (20 female, 11 male) aged 60-98, have taken part in the study to date. They were recruited either through an NHS hospital (16) or third-sector organisations (15) in London and Manchester. The inclusion criteria included anyone over 60 who self-classified as having assisted living needs. Participants were purposively selected to present a range of health conditions and social care needs, including mobility needs, visual/hearing impairment, chronic illness, cognitive impairment, mental health problems and social isolation. Participants were also selected to present a range of socio-economic, cultural and family settings.

The study included an initial sample of 31 participants. However, three of the participants could not carry out the third home visit involving the interview with the Home and Life Scrapbook. Table 1 summarises the profiles of the remaining 28 participants. The sample included a range of health issues, including heart disease, stroke, chronic obstructive pulmonary disease, diabetes, Alzheimer’s disease, falls, visual impairment and osteoarthritis. Seven participants were non-English speaking (three spoke Tamil, three Cantonese, and one French), requiring the interviews and Home and Life Scrapbook materials to be translated.

**Table 1:** Summary of participant characteristics (all names are pseudonyms)

**Participant**	**Sex**	**Age**	**Ethnicity**	**Language**	**Main diagnoses/symptoms**
Rhoda	F	75	White British	English	COPD; Heart disease; Type 2 diabetes; Sleep apnoea
Vera	F	85	White British	English	Stroke; Falls
Nadine	F	90	Asian other	French	Stroke (left sided weakness); Osteoporosis
Ravanan	M	76	Asian other	Tamil	Type 2 diabetes; Hormone deficiency; Neck, shoulder and back pain
Thennan	M	74	Asian Indian	Tamil	Type 2 diabetes; Pain (back, legs)
Tisha	F	72	Asian Indian	Tamil	Heart disease; OA; Type 2 diabetes; Obesity
Colin	M	82	White British	English	Parkinson’s disease; OA; urinary incontinence; falls; gout
Anne	F	84	White British	English	Alzheimer’s disease
Jasmine	F	71	Black Caribbean	English	Stroke (old); Falls; Anxiety and panic attacks; Pain (back)
Pierre	M	73	Black African	English	Falls; Dizziness; Impaired vision, OA; Pain (shoulder)
George	M	90	Black Caribbean	English	Falls; Hypertension, Weakness in legs; Mild cognitive impairment
Geraldine	F	98	White British	English	Alzheimer’s disease
Bilal	M	70	Asian Pakistani	English	Stroke; OA
Elsie	F	82	White British	English	COPD; OA; Falls
Feng	M	82	Asian Chinese	Cantonese	High blood pressure; Impaired hearing
Ping	F	81	Asian Chinese	Cantonese	Pain (arms and legs); Difficulty breathing
Molly	F	77	White British	English	Blind (macular degeneration); High blood pressure
Ruby	F	79	Black Caribbean	English	Visual impairment (blind right eye); OA; Falls; Depression
Neville	M	84	White British	English	Glaucoma; cataract surgery; OA
Betty	F	86	White British	English	OA; Hiatus hernia
Samuel	M	81	Black Caribbean	English	Mild cognitive impairment
Magda	F	88	White Other	English	Mild cognitive impairment; Heart failure; Rheumatoid arthritis; Visual impairment (macular degeneration)
Florence	F	91	White Other	English	OA; Migraine; Leg oedema
Shuang	F	60	Asian Chinese	Cantonese	Depression; Urinary incontinence; Dizziness; Asthma
Ella	F	94	Black Caribbean	English	Falls; OA; Depression
Nina	F	61	White British	English	Diabetes; Pain (back and legs)
Evelyn	F	78	White British	English	Type 2 diabetes; OA; Neck pain
Kate	F	80	White British	English	Polio; Visual impairment;

### Procedure

Each participant was visited on at least three occasions (one participant had a series of shorter visits as this person became easily fatigued). On the first visit, we explained the purpose of the project and asked the participant to consider taking part; we left an information sheet with them. On the second visit, we conducted a semi-structured interview focusing on routines, health, social networks and technology use. At the end of the interview, we presented the Home and Life Scrapbook and camera and went through each suggested activity in turn, emphasising that they could choose which, if any, to complete. On the third visit (approximately one week later) the researcher and participant reviewed and discussed the digital photos and scrapbook content together. Following the interview, we conducted a ‘home tour’, in which the participant showed us different areas of their home to prompt further discussion about what they did and problems they faced.

The Home and Life Scrapbook consisted of an A4 booklet (Arial font size 18) containing seven activities (see Table 2 for a summary of activities) to help capture information on physical, emotional, social and environmental factors related to health and independence at home. 

**Table 2:** Summary of the Home and Life Scrapbook activities

**Activity**	**Description**
Camera	Digital camera to take photos during the week
Maps	Drawings to show relationships with people, places and objects
Lists	Lists of what they like/dislike, what concerns them and what they are comfortable with.
Wishes	Three things they would like to improve or change about their lives
Body outline	Drawing onto a body outline to indicate symptoms or impairments (e.g. pain, discomfort, weakness or decline)
Home plan	Room layouts to indicate spaces and objects related to daily routines and health
Diary	Activities and events they choose to record over one week

Participants were presented with the scrapbook along with a digital camera and a guide booklet to remind them how to use the materials. The first page of the guide read:

“This booklet explains how you might record your thoughts, ideas and things that happen in the next week or two. This will help us find out more about you and your everyday life. But you don’t have to do any of them. You should complete as much or as little as you like.”

#### Digital camera

This activity was included to encourage participants to record significant events and encourage them to think about aspects of the home or outdoors that were important to them. It was emphasised that they could take any pictures they wanted. As has been done in previous studies, suggested photos were included in the guide booklet [9]. These suggestions were intended to indicate the broad scope of images that could be taken, including *“People you meet”, “Special places or things”, “Things you like to see”, “Things you do not like to see”, “Things you enjoy”, “Things that frustrate you”, and “Places and things related to your health”.*

#### Maps

Three separate pages were included for participants to draw a map to indicate their relationships with places, people and objects. Each page included a shadow silhouette in the centre of the page with ‘Me’ written underneath, representing the participant. The instructions were to use this page to draw or write the places/people/objects important to them. Visual materials have previously been used to explore the role of social supports [18]. However, we were also interested to understand how the physical environment had a bearing on participants’ health, independence and quality of life.

#### Lists

Four separate sections were included for participants to write down ‘likes’, ‘dislikes’, things they were ‘comfortable with’ and things they were ‘concerned about’. This was to encourage them to think about the positive and negative aspects of their life. This activity was left open-ended to maximise scope of conversation during the interview.

#### Wishes

Similar to previous work involving older adults with assisted living needs [22], we included a wish list for participants to write down three things they wanted to change or improve about their life. This activity was open-ended in terms of the topics related to each list, but was limited to three items in order to direct the interview towards priority issues. It comprised three sections starting with the phase “I would like…”.

#### Body outline

The outline of a body was included for participants to draw or write on to indicate areas where they experienced symptoms, pain, discomfort or decline. This was included to encourage participants to think about their health and guide discussions about how their health issues disrupted or impeded what they wished to achieve in everyday life.

#### Home plan

A blank page was included for participants to draw various rooms of the home, indicating any objects they used and activities they performed. They were also instructed to indicate health related objects or activities within these rooms. Previous studies have found this approach useful to capture daily routines and meaning attached to domestic environments [14,15]. We included this activity to encourage participants to think about aspects of the domestic setting that had a bearing on their ability to perform daily tasks and manage their health.

#### Diary

*A diary* was included at the back of the booklet to record chosen activities, routines and events during the week. There were seven pages with three sections for morning, afternoon and evening. Similar to the diary prompts used by Pedell et al. [19], each day included the questions: *“What did you like about today?”* and *“How could today have been better?”* These cues were included to encourage participants to reflect on different aspects of the day and think about what events affected them in both positive and negative ways.

## Findings

The main part of this section presents the detailed insights concerning how the Home and Life Scrapbook supported the collection of ethnographic data from people with multiple and diverse physical, cognitive, social and practical needs in the home setting. The findings provided insights into the usefulness of cultural probes to provoke dialogue and elicit information related to features and routines within the domestic environment. Overall, the cultural probe methodology generated rich information to inform how ALTs might fit into the home (or not) and the implications for wider socio-technical networks (lay, commercial and professional) needed to support their effective use. The findings also generated information at a more theoretical level (and from a more critical perspective) on the complex external structures, interactions and interdependencies that complicate the introduction of health and social care technologies into the home. These include the regulatory and legal constraints within which the NHS and social services operate; social expectation of ‘patienthood’ in a technology-supported health service; commercial influences on ALT manufacture, installation and support; and changing social and demographic patterns of informal care relationships. The range of responses to the probes also highlighted that they were more useful in some individual settings than others, and suggested future directions for developing the method.

### Responses to probe activities

There was a heterogeneous response to the Home and Life Scrapbook across cases. Twelve participants did not use the activities. For many this was due to difficulties writing, drawing and holding the camera, caused by physical or sensory impairment (e.g. arthritis severe tremor and impaired vision). Some also felt that they were too fatigued or could not find time to use it (e.g. care demands, stress, managing their health). For example, one participant (Vera) did not use the probe materials due partly to a neurological condition which affected drawing, writing and the use of the camera and partly because she was anxious about numerous hospital appointments and home care packages following a recent fall. In another case, the participant (Nadine) was easily fatigued following a stroke, making it difficult to engage with the probe activities in her own time. However, in all 12 cases these participants underwent the home visit interviews and home tour.

Nine participants chose to use just one activity (Tisha, Bilal, Ruby, Shuang, Neville, Molly, Everlyn, Magda and Nina) themselves or with a family member. This was most commonly the digital camera. One participant (Tisha) used the digital camera but did not complete any activities in the booklet due to literacy problems. Another participant, (Bilal) was initially unable to use the scrapbook and camera due to limited movement in his hands following a stroke. However, he regularly used a touch screen tablet computer, and so this was used to record his ‘wishes’ items.

The remaining seven participants (Rhoda, Ravanan, Thennan, Colin, George, Elsie and Betty) completed nearly all activities, though they varied in how and to what extent they used the probe materials. All used the digital camera, maps and body outline. Six participants completed the diary, lists and wishes. However, only two completed the home plan drawing, which appeared to be too demanding for our participants. Although home plans have been used successfully in previous studies, the activity was a more central component of the research in those cases; fewer additional activities were included and participants did not have multiple physical or cognitive impairments [14,15]. In our study, the home tour appeared to be a more useful and acceptable way to prompt discussion about the use of spaces and objects within the home. On these tours, for example, one participant (Nadine) showed the researcher a jam jar lid, which she had taped over a telecare alarm unit located by the front door. This was done because the alarm button had been repeatedly knocked by people walking through the door, triggering a false alarm. Another participant (Rhoda) expressed negative meanings attached to various health devices in her bedroom. She planned to move the devices behind the bed, out of vision, as they reminded her of her poor health.

### Using the home and life scrapbook

The digital camera was the most commonly used probe activity across the sample. Most participants took a range of photos that conveyed detailed information about the meaningful aspects of the home (e.g. family photos, ornaments, appliances, pets, pictures of deities), routines outside (e.g. walking to church, the park), social encounters (e.g. visitors and club members) and places they visited (e.g. shops, church, temple, community centre). When reviewing images, we asked what was happening in the picture and why the picture had been taken. Reviewing the photos together on the camera display screen felt more like an informal and power-neutral encounter, in which participants typically spoke more openly and freely than in the initial interview. The images themselves also conveyed more information and provided new avenues for conversation.

For example, one Tamil participant (Thennan) captured various images inside his local community centre, including the library, seminar room and staff at the centre. Discussions about the images highlighted the key role of the centre as a social and material resource. When the researcher pointed out the row of computers in the background of one photo, the participant went on to talk about the barriers of learning to use computers. Computer classes were provided at the centre, but he could not fit these around his existing routines, which included managing the home, various time-consuming activities around his own health and also providing daily care and support to his wife. He also commented that he could not ask his children to teach him computer skills as he felt they had their own work and home commitments.

For another participant (Ravanan) the camera acted as a memory aid. During the initial interview, he appeared to have a very limited social network. However, when reviewing his photographs there was a picture of him at a friend’s house. This led to discussion about an enjoyable part of his daily routine in which he and his wife walked the friend’s child to school and the importance of a long-standing friendship with this family.

**Figure 1:**
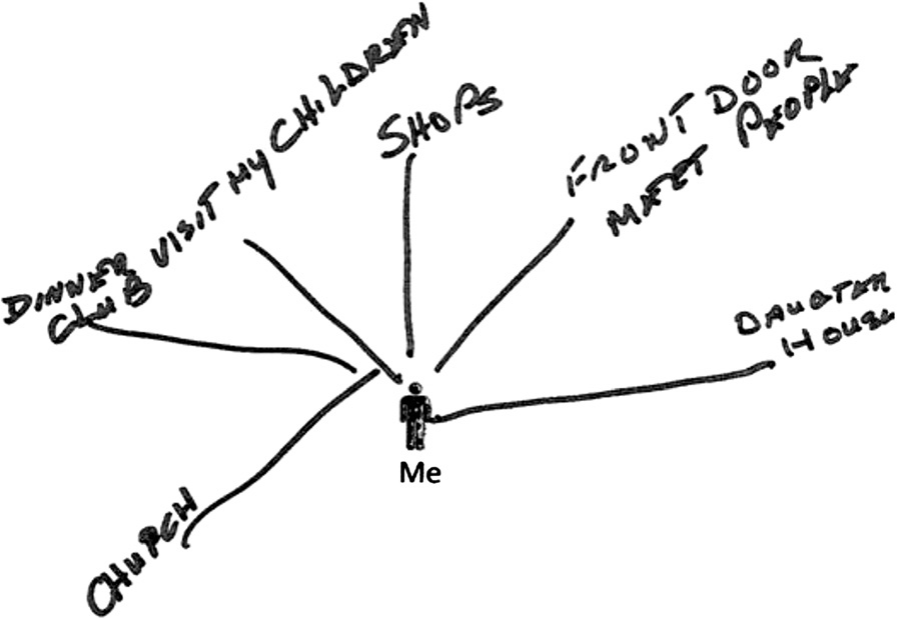
Example ‘places map’ by participant (Rhoda).

The relationship maps prompted participants to reflect on what people, places and objects were important to them outside of the interview setting. One participant (Rhoda) had a number of chronic health conditions and limited mobility. She drew a ‘places map’ that related to locations for face-to-face social interaction (see Figure 1). This included routine visits to the local shop, even if she did not need to buy anything. As she said when reviewing her scrapbook with the researcher: *“It’s my life. I go over there. They all talk to me and, they know me. And I like going over there. It’s my life”.* She also included the ‘front door’ as an important place to greet and chat with people passing by. Since she had very limited mobility she could not venture far beyond her own doorway, but the probe revealed that she spent important periods of time on her doorstep. This led to further discussion of her concerns about moving into sheltered accommodation, where she would not have her own front door or access to local shops as places for opportunistic social interaction. As well as informing a specific ‘implication for design’ – that the user of an ALT may not be as home-bound as designers typically assume – this finding also informed our theorising about the symbolic value of different places and spaces within the home.

**Figure 2:**
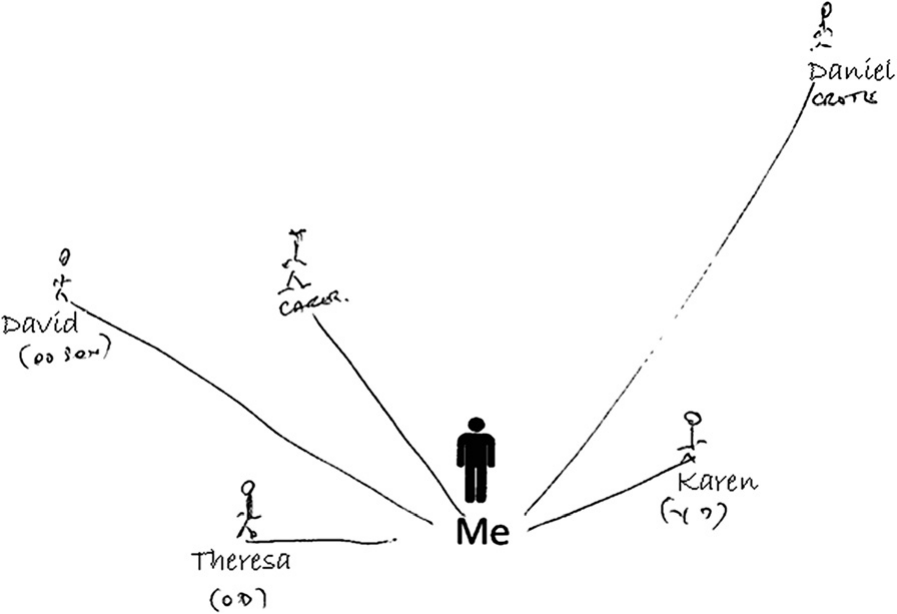
Example ‘people map’ by a participant (Colin) with members of his social support network (names anonymised).

The visual representation afforded by the map component of the cultural probe also helped Rhoda communicate complex relationships. Her ‘people map’ revealed distinctive roles of each individual and the varying types and levels of support they provided. She indicated that one daughter had taken on the main carer role (for example, this daughter undertook daily check-in visits, personal care and supported her to use assistive devices provided by health and social services). Rhoda’s second daughter did not provide instrumental support; instead, her role in the family was to take her out shopping. This daughter also bought gifts for her mother. The third daughter provided limited assistance due to commitments with work and carer responsibilities for another disabled relative. The visual representations drawn by Rhoda helped the researcher follow discussions about complex relationships in more detail, and consider how they related to Rhoda’s health and social wellbeing. Similarly, Colin used the ‘people map’ to represent levels of support by the family. He used proximity from the centre point to indicate geographical distance between his social contacts, which facilitated discussion around the relative levels of support provided by his children (Figure 2).

At a more theoretical level, these finding helped us develop a framework for considering the different roles and routines within the family. Not only do relatives engage in different ways, and at different levels, with an older person’s assisted living needs, but families may develop sophisticated division of labour in this regard which is hard to tease out but which has significant implications for embedding ALTs within the care network. This finding has implications for ALT service providers, particularly with regard to the involvement of informal social networks when providing ALT solutions.

The ‘lists’ activity was included to encourage participants to think about positive and negative aspects of their lives. Its opened-ended nature broadened scope for discussion, highlighting indirect influences on health. For example, Thennan listed *“property at home”* and *“mortgage”* as concerns. During the discussion that followed he described how these worries occupied his mind, distracting him from other domestic and health related activities: *“Because of this I keep forgetting important things, hospital appointment. I go there and they say there was no appointment and I don’t know whether I forgot the previous appointment or not”.* This highlighted how Thennan’s ALT use is overshadowed by significant financial (and resulting emotional) stress, and also at a more theoretical level how ALT acceptance and use must be considered in relation to the social determinants of health and, in particular, the pervasive impact of material poverty.

The lists also guided the interviewer towards key areas for investigation. For example, Colin included issues related to caring for his wife with dementia on his list of concerns. This included the *“wanderings in her head”*, which opened up discussion and led directly into what technologies could support him, and how his existing provision was inadequate. He had a telecare personal alarm provided for his own safety. However, his main concern actually related to the safety of his wife, particularly her risk of falling when getting up during the night. This highlighted the need for detailed assessment and greater personalisation of ALTs provided.

The ‘three wishes’ activity helped explore what participants *wanted*, as well as what they *needed*. Responses ranged from specific issues, such as *“able to breathe better”,* to broader desires, such as *“visiting places”* (remaining active)*, “be able to stay in my own flat”* (ageing in place) and *“able to help orphans”* (reciprocity)*.* Others have previously emphasised the important difference between *needs* and *wants* when designing assistive technology, particularly with regard to the aesthetics of the design [23]. In this study, asking participants to think about what they wanted provided further insight into factors that impacted their quality of life, and opportunities for support. For example, Bilal indicated in his wish list that he would like to be able to go for a walk alone every morning. He had difficulty walking and a fear of falling, and so activity outside was limited to a short walk around his block of flats with a relative. This opened up discussion about his telecare pendant alarm, which only worked within the home. He feared that nobody would help him up if he fell outside, and felt he would benefit more from an alarm that was not limited to indoors.

**Figure 3:**
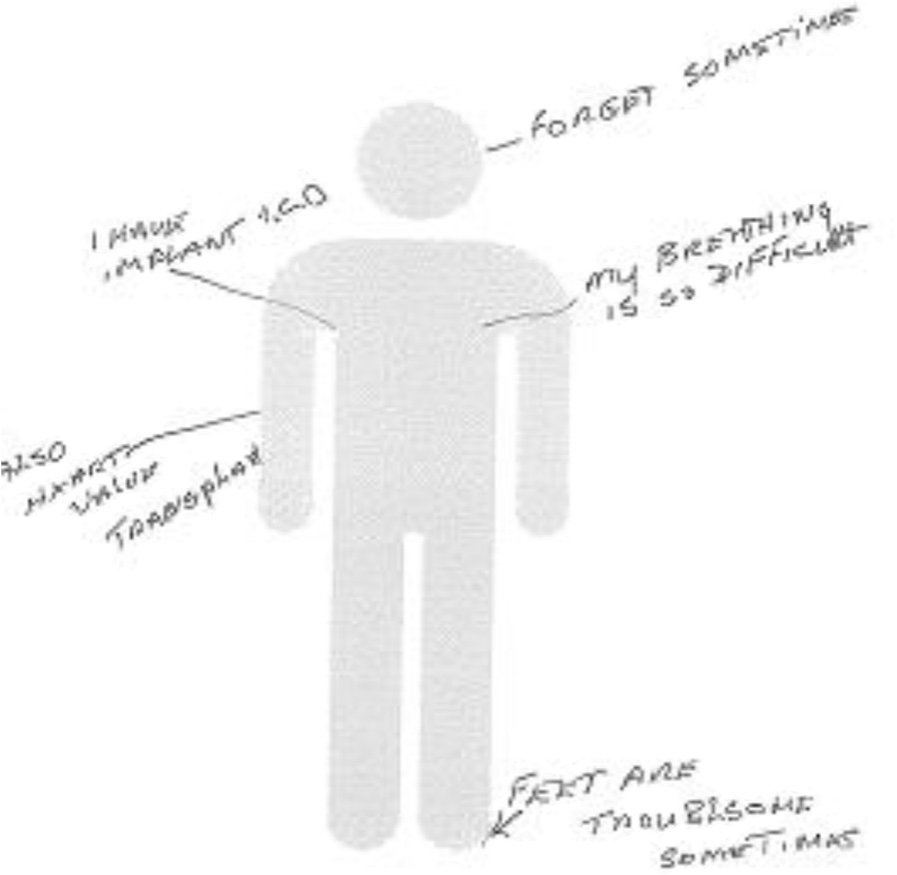
Example ‘body outline’ by a participant (Rhoda).

The ‘body outline’ component of the probe supported discussions around cognitive and physical issues, particularly with participants suffering from multiple conditions. Using the body outline, Rhoda confirmed many of the physical symptoms that had already been discussed in the interview. However, she also indicated being *“forgetful sometime”’* as having an impact on her life (Figure 3). Rhoda’s spontaneous recording of her memory lapse on the body outline provided the researcher with a ready opportunity to discuss this sensitive topic and how it affected Rhoda’s life. She admitted that although she did not have major cognitive problems, occasional memory lapses did cause her additional stress and anxiety (e.g. remembering if she had switched off the cooker or taps once she had gone to bed). In combination with other health issues (e.g. limited mobility and managing diabetes) this mild memory problem caused significant difficulties and concern. Most memory devices focus on prompting prospective memory (e.g. remembering medication or an appointment). In this case, the solution would need to address anxieties related to retrospective memory in order to provide constant reassurance and reduce stress. At a more theoretical level, this prompted us to consider the complex interaction between multiple sub-clinical conditions such as mild memory loss, mild anxiety and various physical limitations on both the design of ALTs and also the support needed to use them.

**Figure 4:**
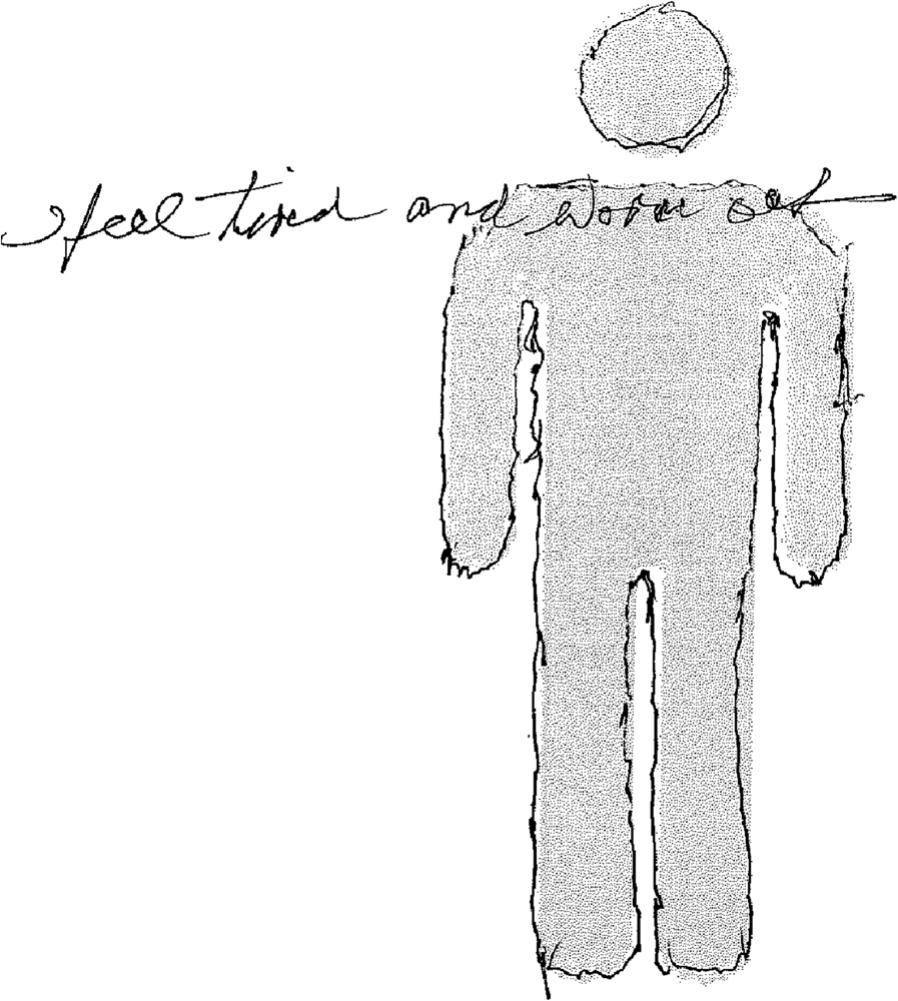
**Example ‘body outline’ by a participant (George).** The text reads “I feel tired and worn out”.

Another participant (George) had drawn along the outside of the body and wrote *“I feel tired and worn out”* across it, which was how he was feeling when using the scrapbook (Figure 4). This opened up a new line of discussion about the occurrence of his low moods, particularly following poor sleep. This guided the interview towards the psychological factors that influenced his independence and quality of life, and how this would change over time. Problems of apathy, or lack of motivation, raise important challenges for embedding ALTs into domestic routines, particularly devices designed to help patients monitor and manage their own conditions and health behaviours. This example also demonstrated the value of using cultural probes to capture subjective accounts and experiences in situ. It is likely that this topic of conversation would not have arisen spontaneously during a conventional research interview, as the participant’s mood had lifted by the time the researcher arrived to his home.

The diaries provided insight into the structure of the day and the importance of daily routines. Diary entries from Ravanan suggested that each day was exactly the same. This was something he said he was happy with and wanted to maintain in order to manage his diabetes. Rhoda’s diary revealed that she had a set morning routine (breakfast, washing, dressing and medication) and evening routine (neighbour visits for chat and television) However, as she had limited mobility and was unable to do housework, it was in the middle of the day that she needed to find things to occupy her time when at home, such as doing crosswords. She commented, however, that these often led to her falling asleep unintentionally, which she believed contributed to her poor sleep during the night. These contrasting examples showed that people may choose to live their lives in a way that accommodates the (sometimes subtle) demands placed on them by chronic illness or disability – and, conversely, that their lifestyle choices may unintentionally exacerbate these illnesses. Analysis of a person’s ‘illness needs’ (and hence ALT needs) cannot meaningfully occur in isolation from their wider living patterns.

The diary entries also captured specific events that would be difficult to obtain through direct questioning in an interview. For example, during the first interview, Colin reported very few difficulties caring for his wife who had dementia. He appeared reluctant to express his concerns face-to-face with the researcher. However, when writing in the diary he described a series of incidents in detail, in which his wife kept getting up during the night. This led to further discussion about how he could benefit from telecare to provide reassurance and peace of mind. One example diary entry read: *“Kept awake most of night, Wife playing about. Keeps getting out of bed….Catheter disconnected 3 times during the night. Not much sleep.”*

The daily prompts (*“What did you like about today?”* and *“How could today have been better?”)* also indicated aspects of the day that had a positive or negative impact. Rhoda added on one of the days that she liked *‘meeting people at the clinic’* during a routine hospital visit. In this case, the social benefit made Rhoda’s trip to hospital a positive experience – a factor which is likely to influence negatively her acceptance of remote assessment. At a more abstract level, this finding prompted us to consider the social as well as biomedical meaning of clinical encounters and the extent to which these are not mirrored in ALT design.

### Cultural probes in use

Literature published to date on cultural probes has tended to focus on the probes themselves, describing the different components and giving examples of design situations in which they were or could be used. These papers have devoted rather less attention to the probe-in-use – for example, they give little detail on the usefulness of these tools or participants’ responses to them [11,12]. This paper documents how the probe materials supported home visit interviews related to features and routines within the domestic environment in order to inform how ALTs, such as telecare and telehealth, might fit into the home.

First, consistent with previous work using cultural probe methods with older adults [21], we found that the probe materials promoted dialogue between researcher and participant, and engaged participants in discussions about their circumstances and needs. Photo sharing was particularly effective in reducing formality, and allowed participants to lead discussions. This was most evident with limited English speaking participants. As visits to these required a translator, it was difficult to establish conversation flow during the initial interview. However, when reviewing the scrapbook the various visual materials and prompts gave them more freedom to set the agenda, elaborate on topics and initiate new lines of conversation. The probe materials were particularly helpful when having conversations with non-English speaking participants through an interpreter.

Second, by keeping the probe materials at home for a week, participants were able to reflect on their daily lives and capture events and experiences in situ. The open-ended nature of the prompts helped broaden scope for discussion. This helped elicit subtle but important issues that would inevitably have been overlooked during an interview. In this respect, it was important that the tool cued people to think both positively and negatively, and not just ask about what problems people had.

Third, the probes acted as a memory aid for participants to recall their personal routines and key events and also capture the subjective elements of these experiences, helping them to participate more effectively in subsequent conversations with the researchers and so rise to the challenge of becoming a ‘co-producer’ of ALTs.

Finally, the visual materials provided a means for ‘cognitive offloading’, helping to communicate complex aspects of daily living. The ‘people map’ was particularly useful in communicating social network structures and helping the researcher follow the participant’s account of members within their network and tease out the various roles and interdependencies of members within it.

## Conclusions

This study has shown that cultural probes are a useful tool to help gain insight into the rhythms, meanings and social influences within the home, and thereby inform the development of ALTs and facilitate a co-production approach to delivering them.

Whilst we have not yet completed a detailed theorisation of our data, the preliminary descriptive findings reported here suggest that cultural probes appear able to assist research that goes beyond technology design and considers (for example) the social determinants of health, the cultural embedding of technology needs and the complex institutional relationships that exist between social actors. Cultural probes appear to have potential to capture small-scale (and seemingly idiosyncratic) details about people’s lives, which can inform a critical ethnography of the macro and meso social structures (economic, cultural/kinship, institutional) within which the lived experience of illness and ageing is inevitably nested. For example, Rhoda’s ‘people map’ and the conversation it prompted about the different roles of her adult children and her concerns about troubling them allowed us to ‘zoom out’ and consider meso-level issues such as the web of relationships within the family and macro-level issues about the social expectations of adult children in contemporary society.

An important finding of this study was that participants with major physical and/or mental impairments, and especially those with multiple co-morbidities, found the use of cultural probes impossible or unacceptable. More work is needed to establish how cultural probe tools can be developed so that they are more accessible to people with physical or sensory impairment (e.g. by using a shortened version with people likely to fatigue easily, or using electronic devices as in the example of Bilal above).

We are now beginning to explore how older people and their families can work directly with ALT sector stakeholders in the co-production of fit-for-purpose ALTs. We anticipate that the future outputs of the ATHENE study will include not only a set of ‘implications for design’ but also a more academic critique and extension of existing theoretical approaches to ALTs.

## Abbreviations

ALT: Assisted living technology; NHS: National Health Service.

## Competing interests

The authors declare that there are no competing interests.

## Authors’ contributions

All authors made substantial contributions to the design of the study JW, PS and SH made substantial contributions to data collection. All authors have made substantial contributions to data interpretation. All authors have read and approved the final manuscript.

## Pre-publication history

The pre-publication history for this paper can be accessed here:

http://www.biomedcentral.com/1471-2288/12/188/prepub

## References

[B1] van Dieren S Beulens JW van der Schouw YT Grobbee DE Neal B The global burden of diabetes and its complications: an emerging pandemic Eur J Cardiovasc Prev Rehabil 2010 17 Suppl 1 S3 S8 20489418 10.1097/01.hjr.0000368191.86614.5a

[B2] Cruz-Jentoft AJ Franco A Sommer P Baevens JP Jankowska E Maggi A Ponikowski P Rys A Szczerbinska K Michel JP Milewicz A Silver paper: the future of health promotion and preventive actions, basic research, and clinical aspects of age-related disease-a report of the European Summit on Age-Related Disease Aging Clin Exp Res 2009 21 376 385 20154507 10.1007/BF03327452

[B3] Greenhalgh T Hinder S Stramer K Bratan T Russell J Adoption, non-adoption and abandonment of an internet-accessible personal health organiser BMJ 2010 341 c5814 10.1136/bmj.c5814 21081595 PMC2982892

[B4] Takahashi PY Pecina JL Upatising B Chaudhry R Shah ND Van Houten H Cha S Croghan I Naessens JM Hanson GJ A randomized controlled trial of telemonitoring in older adults with multiple health issues to prevent hospitalizations and emergency department visits Arch Intern Med 2012 172 773 779 10.1001/archinternmed.2012.256 22507696 PMC3914200

[B5] Steventon A Effect of telehealth on use of secondary care and mortality: findings from the whole system demonstrator cluster randomised trial BMJ 2012 344 e3874 10.1136/bmj.e3874 22723612 PMC3381047

[B6] Greenhalgh T Wherton J Procter R Rouncefield M Dewsbury G ATHENE: Assistive technologies for healthy living in elders: needs assessment by ethnography Proceedings of the digital economy All hands meeting, 15-17 Nov 2011 UK: Newcastle-Upon-Tyne

[B7] Dewsbury G Clarke K Hughes J Rouncefield M Sommerville I Extending home technology design: depending on digital design Hous Stud 2004 19 811 825 10.1080/0267303042000249224

[B8] Hartswood M Procter R Rouncefield M Slack R Voss A Co-realisation: evolving IT artefacts by design Resources, Co-evolution and artefacts 2008 2008th Germany: Springer, Berlin

[B9] Gaver W Dunne A Pacenti E Design: cultural probes Interactions 1999 6 21 29 10.1145/291224.291235

[B10] Boehner K Vertesi J Sengers P Dourish P How HCI interprets the probes Proceedings of CHI’07: 28 April – 3 May 2007 San Jose, USA: ACM 1077 1086

[B11] Graham C Roucefield M Gibbs M Vetere F Cheverst C How probes work Proceedings of OZCHI 2007 2007 Adelaide, Australia: ACM 29 37

[B12] Graham C Rouncefield M Probes and participation Proceedings of participatory design conference 2008 Indiana, USA: ACM

[B13] Dourish P Bell G Divining a digital future: mess and mythology in ubiquitous computing 2011 Cambridge, USA: MIT Press

[B14] Leonardi C Mennecozzi C Not E Pianesi F Zancanaro M Gennai F Cristoforetti A Knocking on elders’ door: investigating the functional and emotional geography of their domestic space Proceedings of CHI’09 2009 Boston, USA: ACM

[B15] Christoforetti A Gennai F Rodeschini G Home sweet home: the emotional construction of places J Aging Stud 2011 25 225 232 10.1016/j.jaging.2011.03.006

[B16] Bernhaupt R Obrist M Weiss A Beck E Tscheligi M Trends in the living room and beyond: results from ethnographic studies using creative and playful probing Comput Entertainment 2008 6 10.1145/1350843.1350848

[B17] Buchmuller S Joost G Sapio B, Haddon L, Mante-Meijer E, Fortunati L, Turk T, Loos E Design for elderly people: a methodological case study about the development of a dect phone The Good, the Bad and the Challenging: The user and the future of information and communication technologies, Conference Proceedings of the Cost Conference 298, 13th – 15th May, Koper 2009 Slovenia: ABS Center 710 719

[B18] Riche Y Mackay W PeerCare: supporting awareness of rhythms and routines for better aging in place CSCW 19 73 104

[B19] Pedell S Vetere F Kulik L Ozanne E Gruner A Social isolation of older people: the role of domestic technologies Proceedings of OZCHI’10 2010 Australia: Brisbane

[B20] Hutchinson H Mackay W Westerlund B Bederson B Druin A Plaisant C Beaudouin-Lafon M Conversy S Evans H Hansen H Roussel N Eiderbäck B Lindquist S Sundblad Y Technology probes: inspiring design for and with families Proceedings of CHI’03 2003 Florida, USA: ACM

[B21] Crabtree A Hemmings T Rodden T Cheverst K Clarke K Dewsbury G Hughes J Rouncefield M Designing with care: adapting cultural probes to inform design in sensitive settings Proceedings of OzCHI’03 2003 Brisbane, Australia: Ergonomics Society of Australia

[B22] Axelrod L Fitzpatrick G Balaam M Mawson S Burridge J Ricketts I Smith P Rodden T A toolkit to explore lived experience of motivation: when words are not enough Proceedings of PervasiveHealth 2011 2011 Dublin, Ireland

[B23] Newell A Clackson J, Coleman R, Keates S, Lebbon C Inclusive design or assistive technology Inclusive design: design for the whole population London: Springer Verlag 172 181

